# Evolution and Diversity of the Antimicrobial Resistance Associated Mobilome in *Streptococcus suis*: A Probable Mobile Genetic Elements Reservoir for Other Streptococci

**DOI:** 10.3389/fcimb.2016.00118

**Published:** 2016-10-07

**Authors:** Jinhu Huang, Jiale Ma, Kexin Shang, Xiao Hu, Yuan Liang, Daiwei Li, Zuowei Wu, Lei Dai, Li Chen, Liping Wang

**Affiliations:** ^1^Laboratory of Veterinary Pharmacology and Toxicology, College of Veterinary Medicine, Nanjing Agricultural UniversityNanjing, China; ^2^Department of Veterinary Microbiology and Preventive Medicine, Iowa State UniversityAmes, IA, USA; ^3^Department of Pharmacy, The Second Hospital of Dalian Medical UniversityDalian, China

**Keywords:** antimicrobial resistance, mobilome, ICEs, MGEs, prophages, *Streptococcus*, evolution

## Abstract

*Streptococcus suis* is a previously neglected, newly emerging multidrug-resistant zoonotic pathogen. Mobile genetic elements (MGEs) play a key role in intra- and interspecies horizontal transfer of antimicrobial resistance (AMR) determinants. Although, previous studies showed the presence of several MGEs, a comprehensive analysis of AMR-associated mobilome as well as their interaction and evolution has not been performed. In this study, we presented the AMR-associated mobilome and their insertion hotspots in *S. suis*. Integrative conjugative elements (ICEs), prophages and tandem MGEs were located at different insertion sites, while 86% of the AMR-associated MGEs were inserted at *rplL* and *rum* loci. Comprehensive analysis of insertions at *rplL* and *rum* loci among four pathogenic *Streptococcus* species (*Streptococcus agalactiae, Streptococcus pneumoniae, Streptococcus pyogenes*, and *S. suis*) revealed the existence of different groups of MGEs, including Tn5252, ICE*Sp*1108, and TnGBS2 groups ICEs, Φm46.1 group prophage, ICE_ICE and ICE_prophage tandem MGEs. Comparative ICE genomics of ICE*Sa*2603 family revealed that module exchange and acquisition/deletion were the main mechanisms in MGEs' expansion and evolution. Furthermore, the observation of tandem MGEs reflected a novel mechanism for MGE diversity. Moreover, an *in vitro* competition assay showed no visible fitness cost was observed between different MGE-carrying isolates and a conjugation assay revealed the transferability of ICE*Sa*2603 family of ICEs. Our statistics further indicated that the prevalence and diversity of MGEs in *S. suis* is much greater than in other three species which prompted our hypothesis that *S. suis* is probably a MGEs reservoir for other streptococci. In conclusion, our results showed that acquisition of MGEs confers *S. suis* not only its capability as a multidrug resistance pathogen, but also represents a paradigm to study the modular evolution and matryoshkas of MGEs.

## Introduction

There has recently been a worldwide rapid emergence of antibiotic-resistant streptococcal infection. In 2013, the US Centers for Disease Control and Prevention (CDC) declared the top 18 drug-resistant threats in the United States, including the “Serious” level drug-resistant *Streptococcus pneumoniae*, and the “Concerning” level erythromycin-resistant *Streptococcus pyogenes* and clindamycin-resistant *Streptococcus agalactiae* (CDC, 2013). *Streptococcus suis* is a previously neglected, newly emerging multidrug-resistant zoonotic pathogen that causes meningitis, septicemia, and arthritis in humans, and is one of the major pathogens that led to substantial economic losses in the intensive swine industry (Lun et al., 2007; Gottschalk et al., 2010). Outbreaks of human *S. suis* infections in China, of which strains were resistant to tetrecyclines and aminoglycosides, in 2005 posed a large public health challenge (Tang et al., 2006; Yu et al., 2006). Previous studies have suggested that *S. suis* is an important antimicrobial resistance (AMR) reservoir that can contribute to the spread of resistance genes to the above-mentioned streptococci (Palmieri et al., 2011; Huang et al., 2016).

Increasing resistance of streptococci to commonly used antimicrobials including tetracyclines (up to >90%) and macrolides (up to >70%) have been reported worldwide since the 1980s (Aarestrup et al., 1998; Zhang et al., 2008; Palmieri et al., 2011), and a number of AMR genes have been identified (http://faculty.washington.edu/marilynr/) (Roberts, 2005; Palmieri et al., 2011). Previous studies reported that the dissemination of these AMR genes in streptococci are associated with different mobile genetic elements (MGEs) (Roberts and Mullany, 2009; Varaldo et al., 2009). MGEs are ubiquitous among all prokaryotes and play a significant role in horizontal gene transfer (HGT) resulting in intra- and interspecies dissemination of AMR and virulence determinants (Dobrindt et al., 2004; Frost et al., 2005; Juhas et al., 2009; Bellanger et al., 2014). The pool of all genes within MGEs, such as integrative and conjugative elements (ICEs), plasmids, insertion sequences (IS), transposons, prophages, integrons and other genomic islands, are collectively referred to as “mobilome” (Frost et al., 2005).

Several MGEs, carrying AMR determinants for tetracyclines, macrolides, aminoglycosides, and chloramphenicol, have been identified in *S. suis* (Chen et al., 2007; Holden et al., 2009; Li et al., 2011; Palmieri et al., 2011). A *tet*(M)- and *aadE*-carrying 89-kb (89 K) pathogenicity island (PAI), which was found to be unusual in streptococcal toxic shock syndrome (STSS)-causing *S. suis* strains, was responsible for full bacterial virulence in two major outbreaks in China (Tang et al., 2006; Chen et al., 2007; Li et al., 2008). The 89 K shares similarity with conjugation modules of *S. pneumoniae* Tn5253, but its integrase belongs to the ICE*Sa*2603 family which site-specifically integrates at *rplL* site instead at *rbgA* site of Tn5253 (Ayoubi et al., 1991; Li et al., 2011). A *tet*(W)-carrying prophage ΦSsuD.1 was found in a *S. suis* strain isolated from a patient with meningitis and is highly similar with the main *S. pyogenes* Φm46.1 and *S. agalactiae* λSa04 (Brenciani et al., 2010; Palmieri et al., 2010). More recently, chimeric and tandem of ICEs in streptococci have been reported (Yao et al., 2015; Huang et al., 2016), which indicate interaction of MGEs occurs to extend MGE diversity and complexity.

Although, some MGEs have been identified in *S. suis*, the knowledge of the prevalence and diversity of MGEs remain largely underscored. Further, interaction of MGEs and their evolution in streptococci have not been identified. In this work, we present the AMR-associated mobilome and their insertion hotspots in *S. suis* to have a better understanding of *S. suis* resistome. Comparative and evolution analysis were conducted at *rplL* and *rum* loci in *S. suis* as well as *S. pneumoniae, S. pyogenes*, and *S. agalactiae* (in total 6491 genome sequences obtained from GenBank) to explore the diversity and evolution of MGEs. This is the first study that provides the landscape of the prevalence and diversity of MGEs at *rplL* and *rum* loci in *S. suis, S. pneumoniae, S. pyogenes*, and *S. agalactiae* to comprehend the underlying evolutionary mechanism of MGEs. Our work further confirms the hypothesis that *S. suis* is an important AMR reservoir and suggest that *S. suis* might be a MGEs reservoir for other streptococci which promoted the worldwide emergence of antibiotic-resistant streptococcal infection.

## Materials and methods

### Bacterial strains and antimicrobial susceptibility tests

A total of seven *S. suis* isolates from our routine surveillance on antimicrobial resistance were chosen and sequenced in this study as they displayed different resistance genotype (Table S1). The complete genomes of five strains from GenBank database were also analyzed (Table S1). Isolated colonies were grown on Todd-Hewitt broth (Difco Laboratories) supplemented with 5% (v/v) sheep blood and incubated at 37°C. *S. suis* experiments were performed in a Biosafety level 2 laboratory. Erythromycin, tetracycline, streptomycin, and kanamycin were purchased from Sigma-Aldrich Co. LLC., Shanghai, China. Antimicrobial susceptibility tests (MICs) were determined by a standard broth micro dilution method.

### Whole-genome sequencing and amplification experiments

The whole genomes of seven strains were sequenced and assembled on the Roche 454 GS Junior System at the Bioinformatics Center of Nanjing Agricultural University (Nanjing, China). These scaffolds of each genome were ordered according to the reference genome of the *S. suis* strain P1/7 (AM946016), using the Mauve v2.4.0 software (Darling et al., 2004). PCR assays were employed to close the gaps to obtain whole sequences of predicted MGEs.

### Identification of resistance genes and mobile genetic elements

MLST classification was derived directly from WGS data by the Web-based method (Larsen et al., 2012), and further confirmed with traditional PCR method (http://www.mlst.net/). Acquired antimicrobial resistance genes were identified using ResFinder 2.1 (Zankari et al., 2012).

MGEs candidates were roughly located when genomes were compared with *S. suis* reference strain P1/7 by MAUVE 2.4.1 (Darling et al., 2004). Prophages were predicted with Phage_Finder (Fouts, 2006). ICEs were predicted with the present of type IV secretion systems (T4SSs) and integrases (Int). Boundaries and insertion sites of both prophages and ICEs were manually checked. ISs were identified with ISfinder (Siguier et al., 2006). Transposons were identified by comparing candidate regions containing associated transposase genes against the NCBI nucleotide database. Putative insertion sites and *att* sequences were manually identified.

### Analysis of insertions at *rplL* and *rum* loci

To obtain the insertion landscape at *rplL* and *rum* loci, 523 genome sequence of *S. suis* were obtained from GenBank (December, 2015). For *rplL* and *rum* loci, sequences between *rplL* and *hdy* genes as well as *rum* and *glf* genes were analyzed, respectively. Three conditions will be considered: (1) no insertion, sequence length will be ~128 or ~561 bp; (2) with insertion and gap, no sequence will be acquired; (3) with full length insertion, sequence length will be insertion length plus ~128 or ~561 bp. The obtained sequences with insertion were further annotated by an online PATRIC server (Wattam et al., 2014). ISs, prophages, transposons, and ICEs were identified as above mentioned.

### Comparative analysis of ICEs groups

For ICE identification, signature proteins of integrase, relaxase and VirB4 were searched by BLASTp comparison. Filters were defined by the coverage (>25% and cover function domain), *E*-value (1e-5) and length (>300 aa for Int, >150 aa for relaxases, and >320 aa for VirB4). If all three proteins were present, the elements were considered ICEs, elements that only harbored integrase and partially or no relaxase and VirB4 were considered defective ICEs (dICEs). Similar analysis of insertions was also done in 637 *S. agalactiae*, 5063 *S. pneumoniae*, and 268 *S. pyogenes*.

Phylogenetic tree of integrases, relaxases and VirB4 proteins were further analyzed. Protein identity of less than 60% was considered as different clade. ICEs were classified by the presence of signature integrases, relaxase, VirB4, and AMR profiles.

Two-hundred and ten ICE*Sa*2603 families of ICEs were retrieved from insertions at *rplL* and *rum* sites. BLAST atlas maps were built by comparing ICEs with reference sequences of 89 K (CP000407) using BLASTn with a >80% identity threshold within the CGView Comparison Tool (Grant et al., 2012).

### Conjugation assay

In mating experiments, two derivative strains *S. suis* P1/7RF (The same as BAA-853RF) and SS-1RF (tetracycline- and erythromycin-susceptible but rifampin- and fusidic acid-resistant) were used as recipient and *S. suis* strains (tetracycline- and erythromycin-resistant but rifampin- and fusidic acid- susceptible) which harbored different MGEs were utilized as donors. Filter mating assays were performed as previously described (Li et al., 2011). The transconjugant was further confirmed by PCR, sequencing, and MLST typing.

### Fitness measurements

The fitness difference between the MGEs-carrying clinical isolates and the reference strain P1/7 were calculated by *in vitro* growth and competition assay. *In vitro* competition, culture of each competitor was adjusted to OD_600_ = 0.2, mixed in a 1:1 ratio, and diluted 1:100 in 5 mL for 10 days. The cfu of the competitors was counted by plating onto medium with or without erythromycin and tetracycline. The relative fitness (*w*) was determined in competition experiments in triplicate and repeated at least twice, as previously described (Starikova et al., 2013).

### Data analysis

Statistical analysis was performed and illustrated with GraphPad Prism 5 (GraphPad Software, Inc.). Insertions analysis of the prevalence of each MGEs groups in these four species and statistics of growth rate and relative fitness (*w*) in fitness assay were analyzed using student *t*-test. A *P* < 0.05 was considered statistically significant.

### Nucleotide sequence accession number

The Whole Genome Shotgun (WGS) sequence of YY060816 has been deposited at GenBank under the accession number AYSB00000000. Sequences of 17 MGEs identified have been deposited at GenBank (Accessions: KX077882-KX077898).

## Result

### AMR-associated mobilome in *S. suis* isolates

To gain insight into the role of MGEs in dissemination of antimicrobial resistance, seven representative isolates were sequenced. The draft genomes were ordered and compared with reference strain P1/7. Finally, we identified 17 intact MGEs in seven strains including six ICEs, eight prophages, two ICE_prophage type tandem composite mobile genetic elements (here assigned as CMGEs), and one ICE_ICE type tandem ICEs (Figure S1 and Table S1). MLST type, serotype, and acquired AMR genes of the isolates are listed in Table S1. AMR genes mediating resistance to macrolides [*erm*(B), *mef* (A), and *msr*(D)], tetracyclines [*tet*(O), *tet*(40), *tet*(L), *tet*(S), *and tet*(W)], and aminoglycosides [*aph*(3′)-III, *sat4, aac*(6′)-*aph*(2″), and *aadE*] were carried by corresponding MGEs (Table S1).

**Figure 1:**
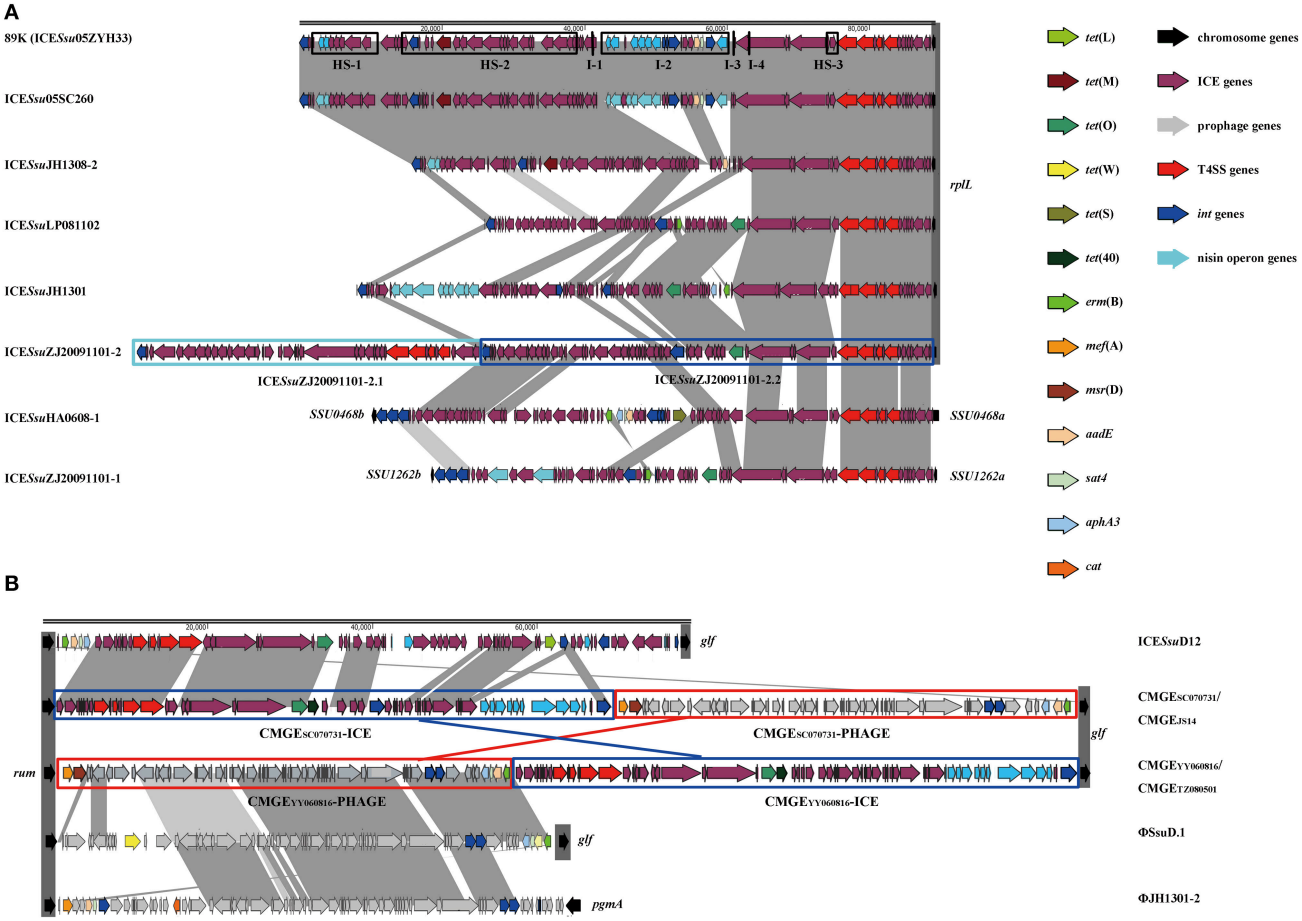
**Genetic context of MGEs at ***rplL*** (A) and ***rum*** (B) loci in ***S. suis*****. ORFs in different color were listed representing different functions. Each part of tandem MGEs were highlighted in color boxes. **(A)** The 89 K PAI were used as references. Accession genes were highlighted in black box with Hotspot (HS) and Insertion (I) as previously described (Huang et al., 2016). ICE*Ssu*JH1308-1 and ICE*Ssu*ZJ20091101-1 inserted in *SSU0468* (*lys*S) and *SSU1262* (*mut*T) were also indicated. **(B)** Multiple MGEs were inserted in 3′ of *rum* gene.

The size, GC content, insertion site, and insertion sequence of each MGEs were further analyzed (Table S1). ICEs were usually integrated at 3′ terminal of the *rplL* gene with the exception of ICE*Ssu*JH1308-1 (in *SSU0468*) and ICE*Ssu*ZJ20091101-1 (in *SSU1262*) (Figure 1A). Conserve CDs of ICEs including the modules of integration/excision (Int and Xis) and conjugation (relaxase and T4SS proteins) are presented in ICE*Ssu*05SC260, ICE*Ssu*JH1308-2, ICE*Ssu*LP081102, and ICE*Ssu*JH1301 (Figure 1A), which can be classified into ICE*Sa*2603 family according to the ICE core structure (conjugation modules; Bi et al., 2012). Three integrases of ICE*Ssu*JH1308-1 and ICE*Ssu*ZJ20091101-1 belonged to the serine family of recombinase instead of the tyrosine family recombinase of Int_ICE__*Sa*__2603_ (Figure 1A and Table S1).

**Figure 2:**
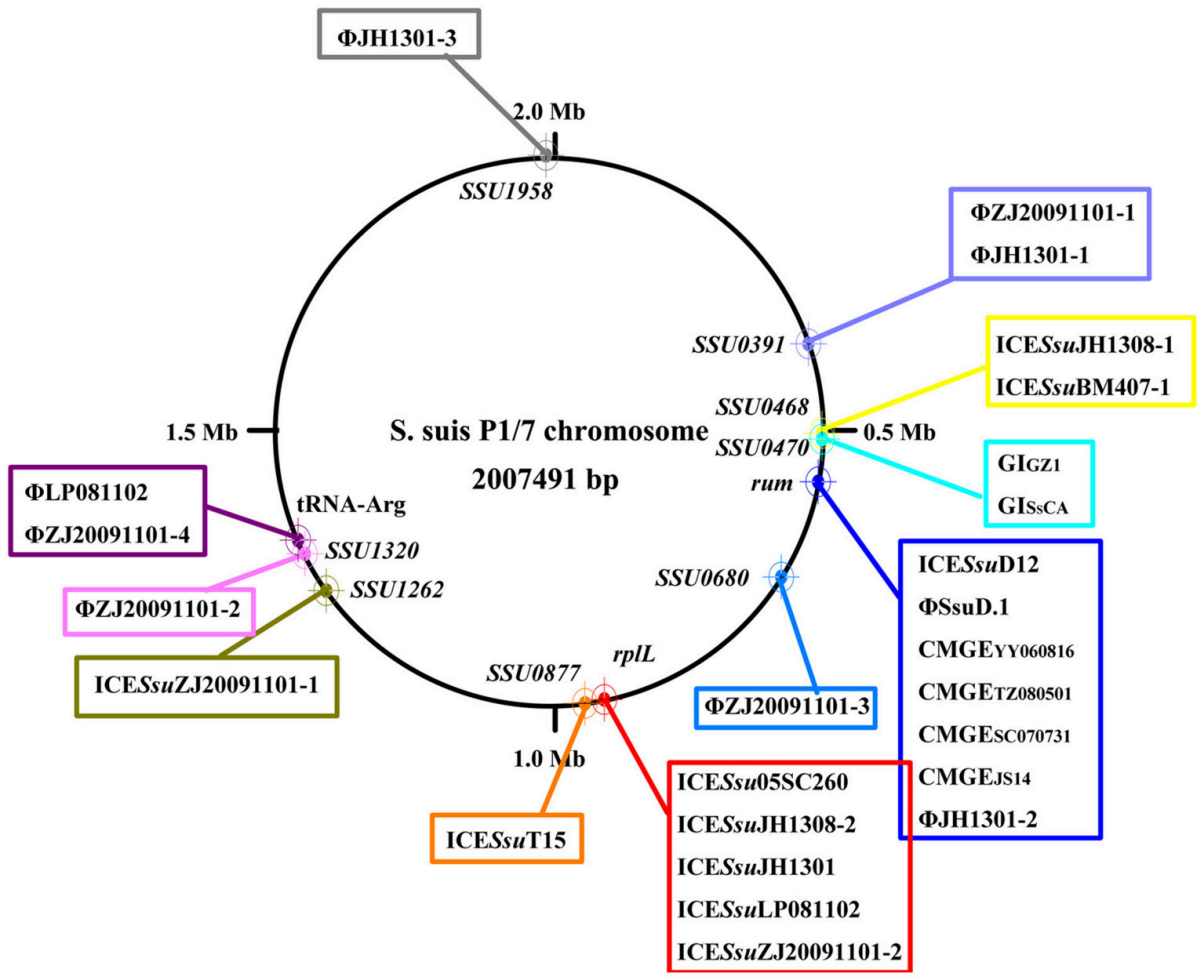
**Schematic map of insertion sites of MGEs identified in this study and previously reported (Palmieri et al., 2011)**. Insertion sites were mapped to reference strain P1/7.

Prophages are known to have increased diversity, thus we chose to only focus on AMR-associated prophages in this study. The *mef* (A)-carrying prophages ΦJH1301-2 and two CMGEs (CMGE_YY060816_ and CMGE_TZ080501_) were inserted in the 3′ terminal of *rum* gene (Figure 1B). CMGE_SC070731_, CMGE_JS14_, ICE*Ssu*D12, and ΦSsuD.1 (Palmieri et al., 2010) identified from publicly-available genomes were also found inserted in *rum* gene (Table S1). A scheme of insertion site of the MGEs to the genome of reference strain P1/7 is shown in Figure 2. These results suggested that *rplL* and *rum* loci are major insertion hotspots for integration of MGEs in *S. suis*.

### Insertion analysis at *rplL* and *rum* loci

As the data thus far had shown that MGEs inserted at *rplL* and *rum* loci were the main MGEs responsible for antimicrobial resistance in *S. suis*, to further obtain the prevalence and diversity of MGEs and their interaction, 523 genome sequences of *S. suis* strains were retrieved from GenBank and analyzed to identify MGEs. At *rplL* site, 326 (62.32%) of the strains contained insertions. Among them, 194 (59.51%) were fully sequenced with no gap between *rplL* and *hdy* genes (Table S2A). However, at *rum* site, the number of strains containing insertions and fully sequenced with no gap were 201 (38.43%) and 73 (36.32%), respectively (Table S2B). The isolation data, insertion length, MGEs profiles, and AMR genes are summarized in Table S2. ICEs, prophages, and tandem MGEs were identified at *rplL* and *rum* loci, but ICEs were the majority of MGEs in both loci.

### Classification of ICE groups

**Figure 3:**
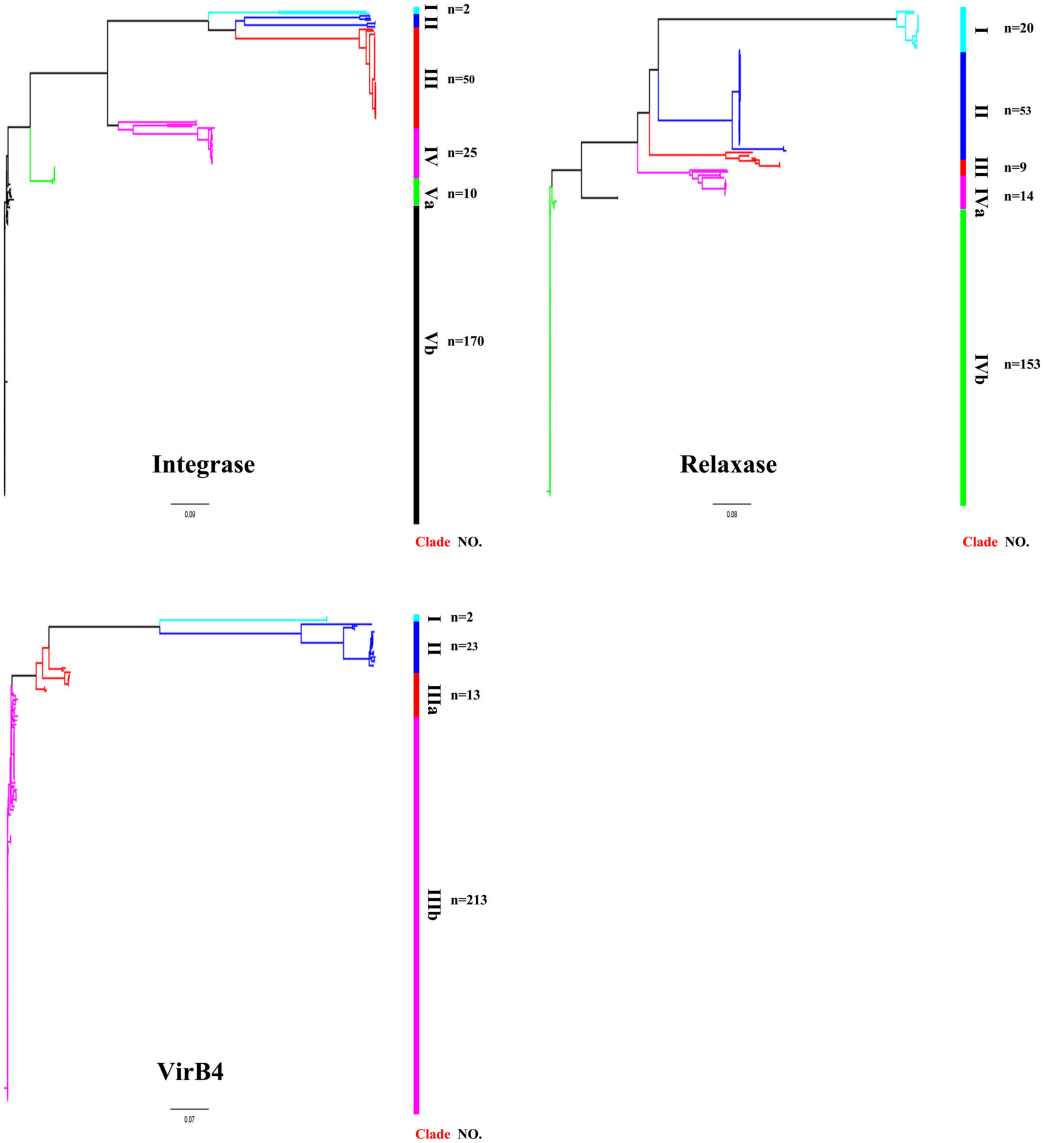
**Phylogenetic tree of integrases, relaxases, and VirB4 proteins**. Protein sequences were aligned by Clustal Omega and Neighbor-Joining tree was constructed. Sequence identity less than 60% were classified into different Clades. The Clade and number of isolates were shown in right of the tree. For detail information, see Tables S2, S3.

ICEs display a modular structure which had previously been analyzed as the modules of integration/excision, conjugation, and adaption (Bellanger et al., 2014). Herein, signature proteins of integration/excision (integrase) and conjugation (relaxase and VirB4) modules were searched by BLASTp comparison. In total, 265 serine or tyrosine family integrases, 252 relaxases, and 253 VirB4 proteins were detected. Phylogenetic analysis showed that integrases, relaxases, and VirB4 proteins could be classified into five, four, and three distinct clades, respectively (Figure 3). Further domain feathers of each clade/sub-clade are shown in Table S3. Clade I-III integrases had specific integrates in *rum* site belonging to serine family recombinase. Clade IV and V integrases belonged to tyrosine family integrases and specifically integrated at *rplL* site. According to the CONJscan-T4SSscan (Guglielmini et al., 2011), all relaxases and VirB4 proteins belonged to MOBp and VirB4 family, respectively. However, the 20 Clade I relaxases that contain a “Relaxase” domain (Pfam03432) were more distant than other groups which also contained an additional C-terminal “Streptin-Immun” domain (Pfam11083). All VirB4 proteins contained a unique “AAA_10” domain (Pfam12846) but belong to three distantly related Clades (Figure 3).

**Figure 4:**
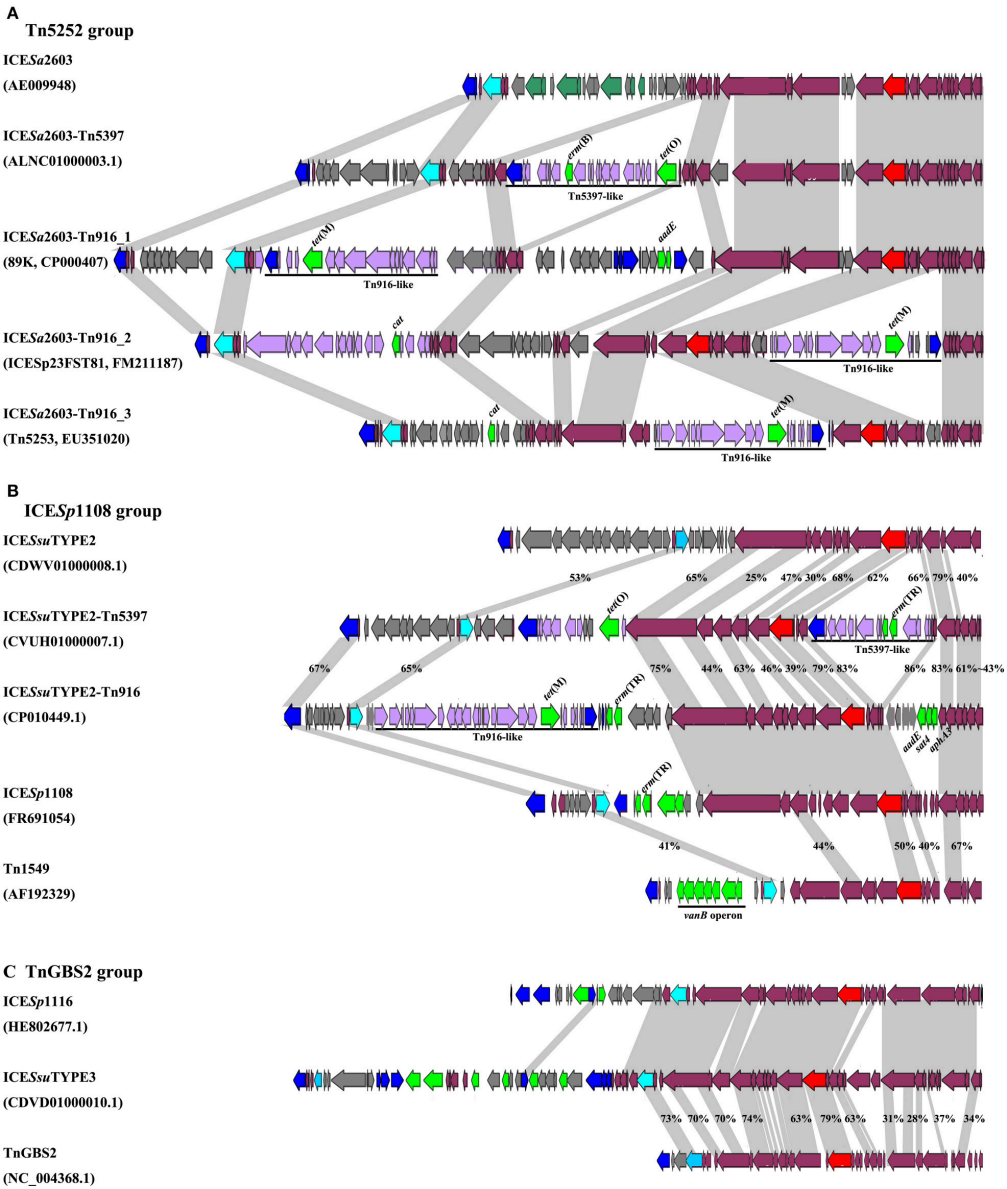
**Schematic diagrams of three groups of ICEs inserted at ***rplL*** and ***rum*** loci**. Int, relaxase, VirB4, and AMR determinants were indicated in blue, light blue, red, and green, respectively. MGEs integrated were highlighted in black straight line. ORFs identity was shown in light gray shadow and identity less than 90% were indicated. **(A)** Comparisons of Tn5252 groups. The prototype ICE Tn5253 sequence was retrieved from NCBI under accession EU351020. Tn5253 integrated at *rbgA* site. **(B)** Comparisons of ICE*Sp*1108 group. This group of ICEs was previously classified into Tn1549 group, but this study showed different genetic context thus was classified as ICE*Sp*1108 group. **(C)** Comparisons of TnGBS2 group. ICE*Ssu*TYPE3 is highly identical with ICE*Sp*1116 in core structure, a TnGBS2 family ICEs.

VirB4, the most conserved protein in conjugation module, is used in the classification of ICE groups (Guglielmini et al., 2011), which we also performed in this study. In addition to ICE*Sa*2603 family, we identified two novel types of ICEs, designated ICE*Ssu*TYPE2 and ICE*Ssu*TYPE3, based on phylogenetic trees of VirB4 proteins and followed by analysis of the core conjugation module (Figure 4). We also performed the occurrence of integrases and relaxases within each types of ICEs to evaluate the modular evolution of ICEs. Table 1 summarizes the co-occurrence of integrases, relaxases, VirB4 proteins, and AMR profiles in ICE groups. Further comparison of ICEs to other known streptococcal ICEs allowed us to reclassify ICEs into three groups named by their prototype: Tn5252, ICE*Sp*1108, and TnGBS2, respectively (Figure 4).

**Table 1:** **Characterization of the ICEs and tandem MGEs at ***rplL*** and ***rum*** loci in ***S. suis*****.

**MGE groups**	**NO**.	**Int^a^**	**Relaxase^b^**	**VirB4^c^**	**Integrated ICEs**	**AMR Profiles**
**TN5252 GROUP**						
ICE *Sa*2603_rplL	5	Clade Vb	Variable	Clade IIIb	–	Variable
	1	Clade Va	Clade IVa	Clade IIIa	–	–
ICE *Sa*2603_rum	1	Clade III	Clade II	Clade IIIb	–	*aph*(3′)-III, *sat4, aadE, erm*(B), *tet*(O), *tet*(L)
ICE*Sa*2603-Tn5397_rplL	9	Clade Vb	Variable	Clade IIIb	Tn5397	*tet*(O), *erm*(B)^d^
	3	Clade Va	Clade IVa	Clade IIIa	Tn5397	*tet*(O)
ICE*Sa*2603-Tn5397_rum	44	Clade III	Clade II	Clade IIIb	Tn5397	*erm*(B), *tet*(O)
	1	Clade III	Clade IVa	Clade IIIa	Tn5397	*tet*(O)
ICE*Sa*2603-Tn916_rplL	144	Clade Vb	Clade IVb	Clade IIIb	Tn916	*aadE, tet*(M)
ICE*Sa*2603-Tn916_rum	2	Clade III	Clade II	Clade IIIa	Tn916	tet(M)
**ICESP1116 GROUP**						
ICE*Ssu*TYPE2_rplL	6	Clade IV	Clade I	Clade II	–	–
ICE*Ssu*TYPE2_rum	1	Clade I	Clade I	Clade II”	–	–
**TNGBS2 GROUP**						
ICE*Ssu*TYPE3_rplL	4	Clade Vb	Clade III	Clade I	–	*tet*(W), *tet*(L), *erm*(B), *lnu*(B), *aadE, cat*
**TANDEM MGES**						
ICE*Ssu*TYPE2_ICE*Sa*2603-Tn5397	6				Tn5397	*tet*(O)
ICE*Ssu*TYPE2_ICE*Sa*2603-Tn916	1				Tn916	*aadE, tet*(M)
ICE*Sa*2603-Tn5397_ICE*Ssu*TYPE2	2				Tn5397	*erm*(B), *tet*(O)
ICE*Sa*2603-Tn916_ICE*Ssu*TYPE2	5				Tn916	*aadE, tet*(M)
ICE*Ssu*TYPE2_Φm46.1-like	1				–	*mef*(A), *msr*(D)
ICE*Sa*2603-Tn5397_Φm46.1-like	2				Tn5397	*aph*(3′)-III*, sat4, aadE, erm*(B)*, mef*(A)*, msr*(D)*, tet*(O)*, tet*(40)
Φm46.1-like_ICE*Sa*2603-Tn5397	2				Tn5397	*aph*(3′)-III*, sat4, aadE, erm*(B)*, mef*(A)*, msr*(D)*, tet*(O)*, tet*(40)

a, b, c *For more information of the Int, Relasase and VirB4 clade and ICE types, see Tables S2–S4*.

d *ICESa2603-Tn5397_rplL ICEs can either present erm(B)+tet(O) or tet(O) genotype*.

*“–”Means none*.

The ICE*Sa*2603 family (Tn5252 group) consisted of the largest number of ICEs in both sites. Three subgroups were divided by the chimeric of Tn916 or Tn5397. The ICE*Sa*2603 subgroup contained six ICEs at *rplL* site and one ICE in *rum* site. Five ICEs at *rplL* site encode a Clade Vb Int and a Clade IIIb VirB4, and one ICE encoded a Clade Va Int, a Clade IVa relaxase, and a Clade IIIa VirB4. However, the ICE*Sa*2603_rum contained a Clade III Int, a Clade II relaxase, and a Clade IIIb VirB4. The ICE*Sa*2603-Tn5397 subgroup consisted of 12 ICEs at *rplL* site and 45 ICEs in *rum* site. This group ICEs resulted from the integration of Tn5397 in I-2 site of ICE*Sa*2603 (Figure 1A). The ICE*Sa*2603-Tn916 subgroup contained 144 ICEs at *rplL* site and two ICEs at the *rum* site. Similar to ICE*Sa*2603-Tn5397, ICE*Sa*2603-Tn916 was generated by the integration of Tn916 in I-2 or I-4 site of ICE*Sa*2603 (Figure 4A).

The ICE*Ssu*TYPE2 family (ICE*Sp*1108 group) included six ICEs at *rplL* site and one ICE in *rum* site. ICEs of this group encoded a Clade I relaxase and a Clade II VirB4 associated with a Clade IV (*rplL* site) or Clade I (*rum* site) Int (Table 1). However, the ICE*Ssu*TYPE3 family (TnGBS2 group) was only identified at *rplL* site. ICEs of this group encoded a Clade Vb Int which was identical to Int_ICE__*Sa*__2603_, but the conjugation module, a Clade IV relaxase and a Clade I VirB4, was distinct from those of ICE*Sa*2603 family (Table 1). Notably, the ICE*Sp*1108 group encoded multiple AMR genes including *tet*(W), *tet*(L), *erm*(B), *lnu*(B), *aadE*, and *cat*. The schematic of the genetic organization of the three groups of ICEs are shown in Figure 4.

### Genetic variability and evolution of ICE*Sa*2603 family—role of acquisition/deletion and module exchange

**Figure 5:**
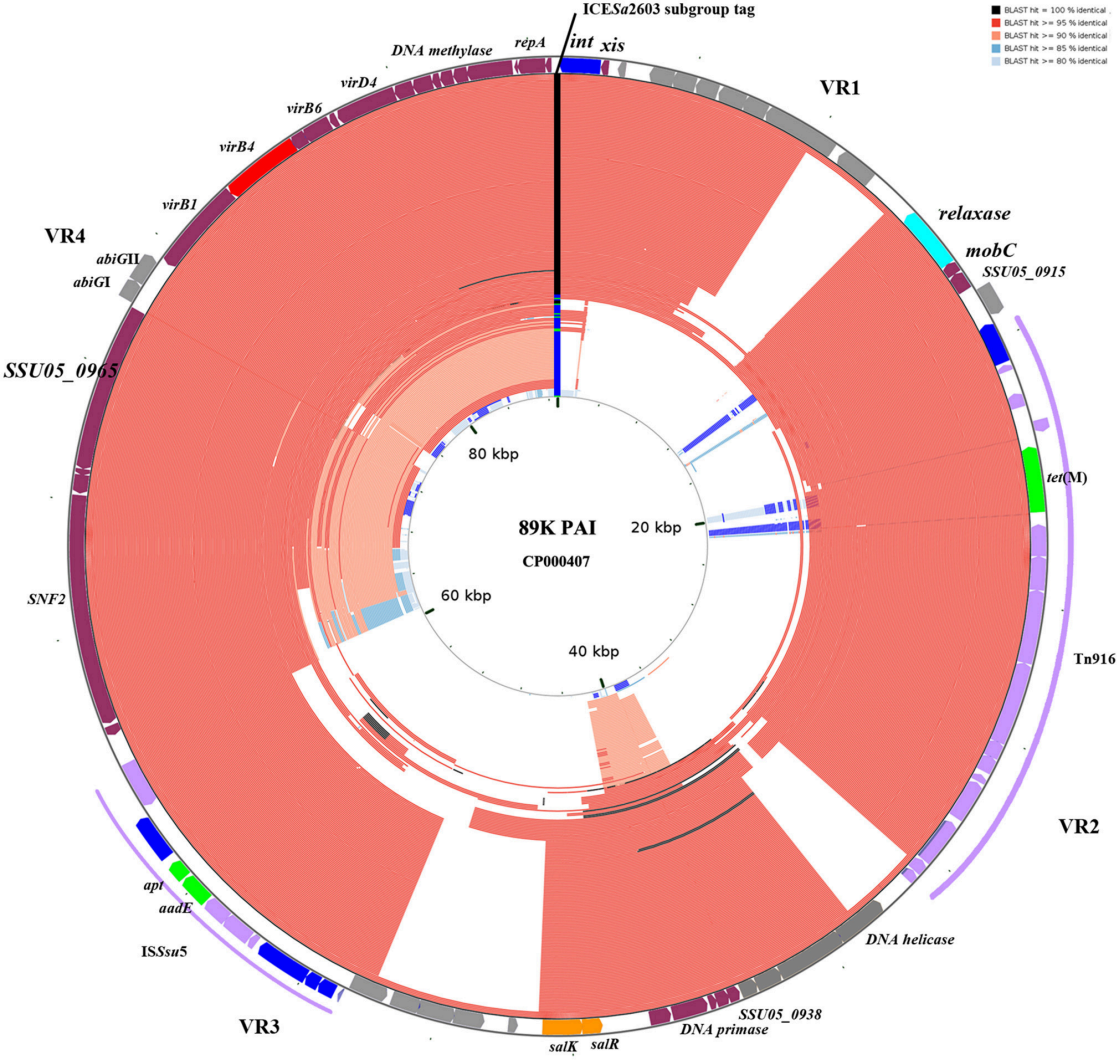
**BLAST atlas of Tn5252 group ICEs**. A total of 210 Tn5252 groups of ICEs were mapped against with the reference sequence of 89 K. ORFs of 89 K are mapped on the most outer circle. Four variable region (VR) are indicated. Different color in ICE*Sa*2603 subgroup tag represent ICE*Sa*2603 (green), ICE*Sa*2603-Tn5397 (blue), and ICE*Sa*2603-Tn916 (black). The 146 ICE*Sa*2603-Tn916 subgroup of ICEs are shown to have alignments at Tn916 region. Five ICEs with Clade IIIa VirB4 were shown in most inner circle. Color gradients are proportional to the BLAST percent identity (80–100%).

The 210 identified ICE*Sa*2603 family ICEs were compared to 89 K reference ICE using BLAST to detect genetic variability and rearrangements within core and variable regions. Figure 5 shows the BLAST atlas for genetic variability of all 210 ICEs against the 89 K.

Genetic variations were mainly observed in four variable regions (VR1-VR4, Figure 5). Gaps of VR1-VR4 in BLAST atlas were due to the different variable-region content of each ICE or absence of a sequence (i.e., the *abi*GI/*abi*GII genes presented in VR4 of some ICEs but absent in other ICEs; Figure 5). The SalR/SalK, which is essential for full virulence of highly invasive *Streptococcus suis* serotype 2 (Li et al., 2008), was only presented in VR3 of ICE*Sa*2603-Tn916 subgroups. Further, acquisition of other MGEs into the ICEs was also observed. The ICE*Sa*2603-Tn916 was composed of the Tn916 insertion in VR2 of the ICE*Sa*2603 structure. An extensive analysis showed that Tn916 could be inserted in different sites which greatly expanded ICE diversity (Figure 4A). Some ICEs of this subgroup even acquired IS*Ssu*5 composite transposon (Figure 5). Deletions were also observed in some genes near variable region probably due to the destruction from recombination/insertion. These results revealed the important role of acquisition of additional genes in ICE diversity and evolution.

**Figure 6:**
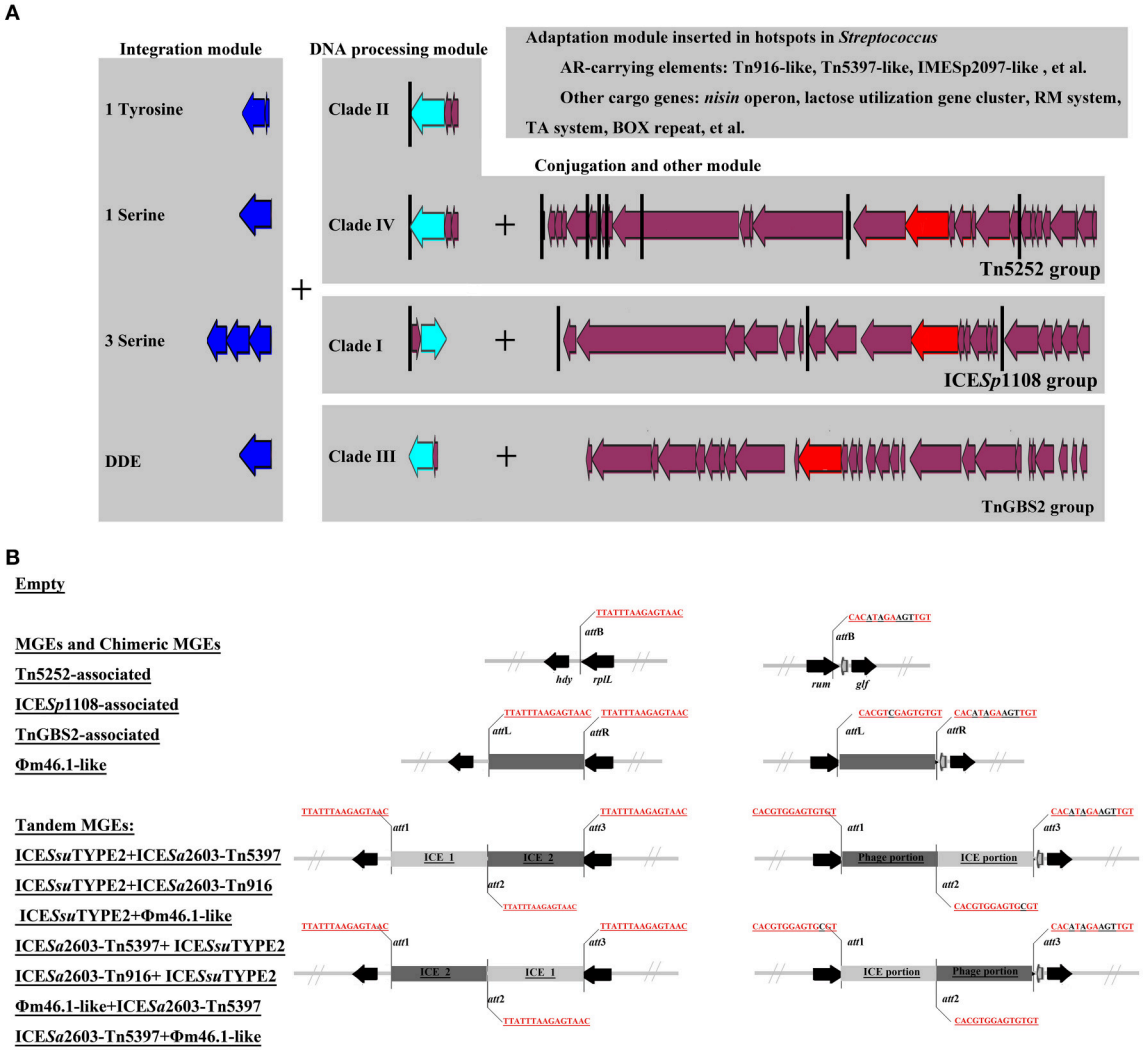
**A schematic model for module exchange and acquisition/deletion (A), and tandem accretion (B). (A)** Int, relaxase, VirB4, and AMR determinants were indicated in blue, light blue, red, and green, respectively. Core conjugation genes were shown in purple. Black vertical line indicates the insertion sites for variable DNA. Exchange of integration module may occur to form different combination of ICEs. **(B)** Schematic map of different types of MGEs and empty form of MGEs at *rplL* and *rum* sites.

The role of module exchange in ICE diversity was evaluated. Most genes in the conjugation module were conserved except three DNA processing genes (*relaxase, mobC*, and *SSU05_0915*). Phylogenetic analysis of relaxases and VirB4 proteins showed different combination in subgroups, which suggested that exchange of DNA processing modules may occur although the evolution of relaxases into different Clade cannot be ruled out (Table 1). Integrases of the 210 ICEs analyzed belonged to tyrosine (at *rplL* site) or serine recombinase (at *rum* site) families but harbor a common conjugation module (Figure 5 and Table S3). The above ICE*Ssu*JH1308-1 and ICE*Ssu*ZJ20091101-1 contained the same conjugation module of ICE*Sa*2603 but encoded three serine recombinases and integrated into different sites (Figure 1A). ICEs encoding the same integration/excision module but with unrelated conjugation modules were also observed in this study (Figure S2). Overall, exchanges of integration/excision module frequently occurred which enhanced ICE diversity and broadened the insertion hotspot for ICE integration. A schematic model for module exchange and sites for integrating of MGEs or other adaptation genes are presented in Figure 6A.

### Tandem accretion—novel mechanism for MGE diversity

An MGE encoding site-specific integrase can integrate into either *att* site of a cell already harboring an MGE thus can form a tandem MGE. By mobilome analysis of clinical *S. suis* isolates above, we identified two types of tandem MGEs, ICE*Ssu*ZJ20091101-2 (ICE_ICE) and CMGE_YY060816_/CMGE_TZ080501_ (ICE_Prophage). Further insertion analysis at *rplL* and *rum* sites identified 17 tandem MGEs displaying various tandem combinations (Table 1). These tandem MGEs typically contained three *att* sites flanking MGEs (Figure 6B). Notably, the phage_ICE (or ICE_phage) CMGE-carrying isolates were identified in different regions of China from the southwest (SC070731, Sichuan) to southeast (TZ081102, Zhejiang). A clinical screen of the coexistence of the CMGEs' core ICE and phage genes showed the percentage as ~11.11% (data not shown), which may underscore a potential dissemination of this novel CMGEs-carrying *S. suis*.

### Transferability and fitness of MGEs

In conjugation assay, four clinical MGEs-carrying strains 05SC260, YY060816, LP081102, and ZJ20091101 were used as donors and strains *S. suis* P1/7RF and SS-1RF were utilized as recipients. However, we only obtained clones of transconjugant carrying ICE*Ssu*05SC260 in both P1/7RF and SS-1RF at a low frequency of ~4.25 × 10^−8^ and 6.1 × 10^−8^, respectively.

We then detected the fitness of these isolates by *in vitro* growth and competition. Figure S3 shows the growth curve of four isolates (05SC260, YY060816, LP081102, and ZJ20091101) and a reference strain P1/7. The growth of YY060816 exhibited ~3.5 h detention and the maximum density was 14.7% lower than P1/7 although there were no differences in growth rate at logarithmic phase (Figure S3A). No significant differences were observed between 05SC260, LP081102, ZJ20091101, and P1/7 (Figure S3A). *In vitro* competition was also performed to determine whether there were fitness differences between those isolates to reference strain P1/7 (Figure S3B); however, no visible fitness cost was observed between different MGE-carrying isolates.

### The prevalence and diversity of MGEs in *S. suis, S. agalactiae, S. pneumoniae*, and *S. pyogenes*

**Figure 7:**
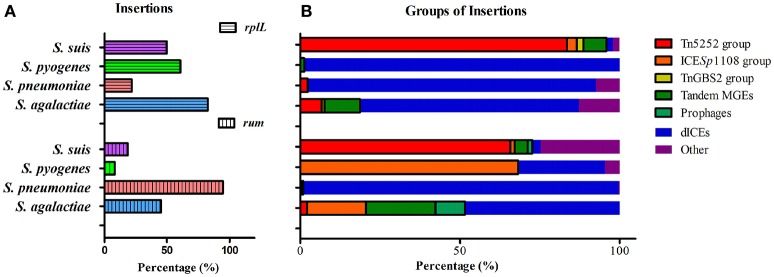
**Prevalence of insertions (A) and percentage of MGEs groups in isolates with insertion (B) in four species at ***rplL*** and ***rum*** sites**.

Since *rplL* and *rum* loci are also the insertion hotspots in other *Streptococcal* species, we further analyzed the insertions in three clinically important species, *S. agalactiae, S. pneumoniae*, and *S. pyogenes*. Tables S4A,B summarizes the isolation data, insertion length, MGEs profiles, and AMR genes of *S. agalactiae, S. pneumoniae*, and *S. pyogenes* isolates obtained from GenBank database. Statistics of the percentage of isolates with insertions within each species is shown in Figure 7A. Among the isolates with insertions, the prevalence of MGEs groups is further shown in Figure 7B. The above-mentioned three groups of ICEs in *S. suis* were also identified in *S. agalactiae, S. pneumoniae*, and *S. pyogenes* which indicated interspecies transfer of MGEs in *Streptococcus* species. The Tn5252 group presented at *rplL* site in *S. suis* (83.51%), *S. agalactiae* (6.78%) and *S. pneumoniae* (2.34%), while at *rum* site the percentage was 65.75% (*S. suis*), 2.06% (*S. agalactiae*), and 0.28% (*S. pneumoniae*), respectively. The ICE*Sp*1108 group presented at *rum* site of *S. pyogenes* (68.18%), *S. agalactiae* (18.56%), *S. suis* (1.37%), and *S. pneumoniae* (0.62%), but at *rplL* site only presented in *S. suis* (3.09%) (Figure 7B and Table S4). The TnGBS2 group only presented in *rplL* site of *S. suis* (2.06%) and *S. agalactiae* (0.85%). Collectively, the prevalence and diversity of MGEs (three groups of ICEs, prophages, and tandem MGEs) is much greater than other three species (Figure 7B).

Unlike ICEs in *S. suis*, in which exchange of integration/excision module and tandem accretion of MGEs play important roles in ICE diversity and evolution, deletion of modules was predominant in ICEs of *S. agalactiae, S. pneumoniae*, and *S. pyogenes* at *rplL* and *rum* sites, which, as a result, generated defective ICEs (dICEs) probably with no function (Table S4). With the exception of *S. pyogenes* at the *rum* site which only had 8.33% (22/268) of the isolates harbor insertions (Figure 7A), the most prevalent MGEs in other species or at other site were dICEs (Figure 7B), including the *S. pyogenes* 10270-RD.1 (Beres et al., 2006; Beres and Musser, 2007), an ICE-like region of difference which contained relaxase and site-specific recombinase but no conjugation module.

## Discussion

We combined our sequenced genomes and publicly-available genomes to identify AMR-associated mobilome and their insertion hotspots in *S. suis*, an emerging drug-resistant zoonotic pathogen that most recently caused two human outbreaks in China (Tang et al., 2006; Ye et al., 2008). ICEs, prophages and tandem MGEs were detected at different insertion sites (Figure 2); but 86% of the AMR-associated MGEs were inserted at *rplL* and *rum* loci (Table S1), two cross-species conserved insertion hotspots in *Streptococcus* and other Gram-positive bacteria (Depardieu et al., 2003; Srinivasan et al., 2014; Marini et al., 2015; Huang et al., 2016). To date, many AMR-associated MGEs have been reported at *rplL* and *rum* sites. At *rplL* site, these include the ICE*Sa*2603 of *S. agalactiae* (Tettelin et al., 2002), the ICE*Sp*23FST81 of *S. pneumoniae* (Croucher et al., 2009), the 10750-RD.1 (dICEs) of *S. pyogenes* (Beres and Musser, 2007), and the 89 K and ICE*Ssu*32457 of *S. suis* (Li et al., 2011; Palmieri et al., 2012). At *rum* site, λSa04 in *S. agalactiae* (Tettelin et al., 2005), Φm46.1 in *S. pyogenes* (Brenciani et al., 2010), and ΦSsuD.1 in *S. suis* (Palmieri et al., 2010) have been identified. However, the prevalence and diversity of MGEs in these species have not been precisely characterized. Moreover, systemic evolutionary analysis involving interaction of MGEs has been lacking in *Streptococcus* species. By analysis of insertions at *rplL* and *rum* loci in 6491 genome of four *Streptococcus* species (Tables S2, S4), we provide the landscape of the prevalence and diversity of MGEs which deepens our comprehension of the underlying evolutionary mechanism of MGEs in *Streptococci*.

Among the identified MGEs, ICEs play a key role in bacterial evolution and adaptation. We classified ICEs among the pathogenic *Streptococcus* species (*S. agalactiae, S. pneumoniae, S. pyogenes*, and *S. suis*) into at least three groups on the basis of their conjugation modules: Tn5252, ICE*Sp*1108, and TnGBS2 (Figure 4). *S. suis* appeared to be the species with the highest rates of ICEs since 88.66% (at *rplL* site) and 67.12% (at *rum* site) of the strains with insertions harbored ICEs, while dICEs were more prevalent at both sites in *S. agalactiae, S. pneumoniae*, and *S. pyogenes* (Figure 7). The Tn5252 group, although its prototype was originally identified in *S. pneumoniae* (Ayoubi et al., 1991), was widely distributed in *S. suis* at both sites, 83.51% and 65.75%, respectively, but a lower rate (< 7%) was shown in *S. agalactiae, S. pneumoniae*, and *S. pyogenes*. The ICE*Sp*1108 group, first reported at *rum* site of *S. pyogenes* (Brenciani et al., 2011), was also present in the other three species at *rum* site but only in *S. suis* at *rplL* sites. Further, TnGBS2 group, tandem MGEs, and prophages are presented in *S. suis* and *S. agalactiae* but not in *S. pneumoniae*, and *S. pyogenes* at both sites (Figure 7). As such, the prevalence and diversity of MGEs in *S. suis* was much greater than other species. Similar results were also observed in a recent study for identification of ICEs in complete genome of *Streptococcus* species (Ambroset et al., 2015). Previously, conjugation transfer of Tn5252 group of ICEs between *Streptococcus* species was reported (Davies et al., 2009; Haenni et al., 2010). Recently, the *S. suis* Tn5252 group of ICE (ICE*Ssu*32457) was shown to have the transferability to *S. agalactiae, S. pneumoniae*, and *S. pyogenes* (Palmieri et al., 2012). Recombination of ICE*Ssu*32457 and *S. agalactiae* ICE*Sa*2603 generated a tandem ICE, which is transferable to *S. pyogenes* strains (Marini et al., 2015). All these observations strengthen the notion that *S. suis* is an important antibiotic resistance reservoir that can contribute to the spread of resistance genes to the above-mentioned streptococci, and is tempting to speculate that *S. suis* is probably a MGEs reservoir for other streptococci.

Our analysis revealed the role of module exchange in shaping ICEs structure and driving its evolution. Exchange of integration module has been reported in several ICEs like SXT/R391 family (Wozniak et al., 2009) and ICE*Sa*2603 family (Huang et al., 2016). However, these analyses only used very few integrases encoded by ICEs and therefore may not be significant. In this study, we showed that Tn5252 groups of ICEs can hijack different types of integrating modules including distinct tyrosine integrases and different number of serine integrases. At least two clades of tyrosine integrases, Int_ICE__*Sa*__2603_ and Int_Tn5253_, were shown in Tn5252 group of ICEs integrating at *rplL* and *rbgA* sites, respectively. Tn5252 group of ICEs with a unique integrase or three serine integrases were observed at *rum* or *SSU1262* (*mut*T) and *SSU0468* (*lys*S) in this study. Although, Tn5252 group of ICEs encodes a DDE transposase was not found in *S. suis*, this type of ICEs may occur during interaction of the ICEs. Further, exchange of integration module also occurred in ICE*Sp*1108 group at least in *S. suis* if not other streptococci (Tables S2, S4). TnGBS2, that originally encodes a DDE transposase was identified in *S. agalactiae* (Brochet et al., 2009), was also recently identified in other streptococci (Guerillot et al., 2013). Our study further confirms an exchange of DDE transposase by Int_ICE__*Sa*__2603_ in TnGBS2 group (ICE*Ssu*TYPE3) in *S. suis* (Figure S2), which also suggests the potential occurrence of Tn5252 group of ICEs with a DDE transposase. Exchange of integration module may also occur between ICEs and prophages since the serine integrase of Φm46.1 phylogenetic resides between that of ICE*Sp*1108 and Tn5252 (Figure 3).

As module exchange plays key role in forming new types of ICEs and expanding the insertion sites for integration, acquisition/deletion of modules play important roles in ICE diversity and adaption. Deletion of conjugation module rarely occurred in *S. suis*, while it was frequently identified in *S. agalactiae, S. pneumoniae*, and *S. pyogenes* (Figure 7). Acquisition/deletion of adaption module frequently occurred in the MGEs studied. Here, we further discussed the ICE*Sa*2603 family (Tn5252 group) in depth to speculate their evolution mechanism. The original host might be *S. suis* since the most majority of ICEs have been identified in this species, including those within other insertion sites (Ambroset et al., 2015). ICE*Sa*2603 at *rplL* site interacts with other MGEs and exchanges the integration module to potentially form new subgroups of ICEs, for example, ICE*Sa*2603_rum, ICE*Sa*2603_mutT, ICE*Sa*2603_lysS. ICE*Sa*2603 can also acquire other MGEs or elements including the *tet*(M)-carrying Tn916, *erm*(B)- and/or *tet*(O)-carrying Tn5397, and *aadE*-carrying IS*Ssu*5 composite transposon generating the “chimeric” or “composite” MGEs: ICE*Sa*2603-Tn5397 and ICE*Sa*2603-Tn916. ICE*Sa*2603, ICE*Sa*2603-Tn5397, and ICE*Sa*2603-Tn916 can undertake exchanging of modules between with MGEs or conjugating transfer to other streptococci. ICEs in their host continue to acquire AMR and virulence genes in order to adapt to the host or degrade into dICEs by deletion of core modules of ICEs. Figure 4A shows the different combination of Tn916/Tn5397 integration in different regions of ICE*Sa*2603 core structure. Notably, by acquiring of SalK-SalR constituent, the *tet*(M)- and *aadE*-carrying 89 K PAI caused two major human outbreaks in China (Li et al., 2008), underscoring the potential spread of ICEs that combination of AMR and virulence determinants.

In addition to module evolution, tandem accretion of MGEs have been reported in *Streptococcus thermophiles* which forms “tandem MGE.” Pavlovic et al. (2004) reported the ICE*St*1 from *S. thermophiles* evolved by deletion and tandem accretion of ICEs and CIMEs resulting from site-specific recombination. A recent study showed that conjugation of *S. suis* ICE*Ssu*32457 and *S. agalactiae* ICE*Sa*2603 generated a tandem ICE, which was transferable between *S. pyogenes* strains under laboratory conditions (Marini et al., 2015). In the present study, our data indicated that tandem accretion with different combination of ICE_ICE and ICE_prophage occurred in *S. suis* (~7%) and *S. agalactiae* (~16%). The prevalence of tandem MGEs in *S. suis* as well as other streptococci indicating that tandem accretion plays a role in evolution of MGEs (Pavlovic et al., 2004; Bellanger et al., 2014).

Acquisition of MGEs was thought to impose an immediate biological cost, the initial costs of MGE carriage may be mitigated during growth (Starikova et al., 2013). The fitness assays indicated no significant fitness cost between MGE-carrying and MGE-free isolates which may be mitigated during transmission. Furthermore, the ICE*Sa*2603-Tn916 group (ICE*Ssu*05SC260 in our study) was shown to be transferable, which was consistent with other studies (Li et al., 2011; Huang et al., 2016). Although, the transfer assay of tandem MGEs was not successful in our study, the transferability of tandem MGEs cannot be excluded because the horizontal transfer of tandem MGEs ICE*St*1 was observed (Bellanger et al., 2009). These results may explain the observation that ICE*Sa*2603-Tn916, ICE*Sa*2603-Tn5397, and tandem MGEs are widely distributed in *S. suis*.

In summary, the study provides an overview of AMR-associated mobilome and their insertion hotspots in *S. suis*, and illustrates the role of module exchange, acquisition/deletion, and tandem accretion in diversity and evolution of MGEs among four pathogenic *Streptococcus* species which suggests that *S. suis* is probably a MGEs reservoir for other streptococci.

## Author contributions

JH, LW developed the concept and designed experiments. JH, JM, KS, YL, and LC performed the experiments and collected the data. JH, XH, and ZW conducted all bioinformatics analyses. JH, DL, LD, and LW prepared the manuscript. All authors have contributed to, seen and approved the manuscript.

### Conflict of interest statement

The authors declare that the research was conducted in the absence of any commercial or financial relationships that could be construed as a potential conflict of interest.

## Supplementary material

Table S1 **Identification of mobile genetic elements identified in the genome of ***S. suis*** strains analyzed in this study**. ^a^Whole genome sequences of MGEs were closed by PCR. ^b^Insertion site genes were checked referring to P1/7. ^c^Putative *att* sites were manually checked by compare of the junction sequences.

Table S2A **Identification of MGEs at ***rplL*** Locus of ***S. suis*****. 523 genome sequence were obtained from genbank. Of the 326 (62.32%) sequences which contain inserts, 194 (59.51%) can obtain non-gap insert sequences. The 194 MGE candidates were further annotated and analyzed for IS, Transposon, phage, and ICEs identification. Int, Relaxase, and VirB4 type and AMR profiles were also analyzed. Strain P1/7 were listed as no insert reference.

Table S2B **Identification of MGEs at ***rum*** Locus of ***S. suis*****. 523 genome sequence were obtained from genbank. Of the 395 (75.53%) sequences which contain inserts, 73 (18.48%) can obtain non-gap insert sequences. The 73 MGE candidates were further annotated and analyzed for IS, Transposon, phage, and ICEs identification. Int, Relaxase, and VirB4 type and AMR profiles were also analyzed. Strain P1/7 were listed as no insert reference.

Table S3 **Classification of Int, Rlaxase, and VirB4**. Sequence identity less than 60% were classified into different Clade. Domain information of each Clade were also indicated. For Int, Clade 1–3 belong to serine recombinase family, Clade 4 and 5 belong to tyrosine recombinase family. Identity between Clade may indicate.

Table S4A **Identification of MGEs at ***rplL*** Locus of ***S. agalactiae, S. pneumoniae***, and ***S. pyogene*****.

Table S4B **Identification of MGEs at ***rum*** Locus of ***S. agalactiae, S. pneumoniae***, and ***S. pyogene*****.

Figure S1 **Comparative genomics of ***S.suis*** isolates and localization of MGEs in genomes**. The scaffolds of each strains was ordered by MAUVE 2.4 with the whole genome sequenced reference strain including P1/7, 05ZYH33, BM407. The ordered genome sequences were further compared with P1/7. Black Box showed the MGEs after manually confirmed.

Figure S2 **Schematic diagram of ICE***Sa***2603-Tn5397_rum, ICE***Sa***2603-Tn916_rplL, and ICE***Ssu***TYPE3_rplL**. Int, relaxase, VirB4, and AMR determinants were indicated in blue, light blue, red, and green, respectively. ORFs identity was shown in light gray shadow and identity of amino acids (aa) was indicated. ICE*Sa*2603-Tn5397_rum and ICE*Sa*2603-Tn916_rplL showed encoding identical conjugation module but different integration module. ICE*Sa*2603-Tn916_rplL and ICE*Ssu*TYPE3_rplL had identical integration module but distinct conjugation module.

Figure S3 *****In vitro*** growth (A) and competition (B) of strain 05SC260, LP081102, YY060816, and ZJ20091101**. Strain 05SC260 carry an ICE*Ssu*05SC260 which is nearly identical to 89K; LP081102 carry an ICE, ICE*Ssu*LP081102, and a prohage, ΦLP081102; YY060816 carry an ICE-phage tandem MGE, CMGE_YY060816_; and ZJ20091101 carry an ICE-ICE tandem MGE, ICE*Ssu*ZJ20091101-2 and 5 other MGEs (See Table S1).
