# Supplementary material for: Evolution and Diversity of the Antimicrobial Resistance Associated Mobilome in Streptococcus suis: A Probable Mobile Genetic Elements Reservoir for Other Streptococci
Source: Front Cell Infect Microbiol. 2016 Oct 7;6:118. doi: 10.3389/fcimb.2016.00118 (PMC5053989; doi:10.3389/fcimb.2016.00118)
Supplement: Table S1 — Identification of mobile genetic elements identified in the genome of S. suis strains analyzed in this study. aWhole genome sequences of MGEs were closed by PCR. bInsertion site genes were checked referring to P1/7. cPutative att sites were manually checked by compare of the junction sequences. [file Table1.DOCX]

**Table S1. Identification of mobile genetic elements identified in the genome of *S. suis* strains analyzed in this study.**

| **Strains** | **MLST** | **Serotype** | **Geographic** | **MGEs ^a^** | **Sizes (bp)** | **GC (%)** | **Acquired resistance genes** | **Insertion site ^b^** | **Putative *att* sequence ^c^** | **Accession** |
| --- | --- | --- | --- | --- | --- | --- | --- | --- | --- | --- |
| YY060816 | ST7 | 2 | Hunan, China | CMGE_YY060816_ | 125,757 | 39.06 | *aph(3’)-III*, *sat4*, *aadE, erm(B)*, *mef(A)*, *msr(D)*, *tet(O)*, *tet(40)* | *rum* | CACGTG**GA**GTGTGT/CACATA**GA**AGTTGT | KX077898 |
| TZ080501 | ST7 | 2 | Zhejiang, China | CMGE_TZ080501_ | 125,779 | 39.05 | *aph(3’)-III*, *sat4*,, *aadE, erm(B)*, *mef(A)*, *msr(D)*, *tet(O)*, *tet(40)* | *rum* | CACGTG**GA**GTGTGT/CACATA**GA**AGTTGT | KX077897 |
| 05SC260 | ST7 | 2 | Sichuan, China | ICE*Ssu*05SC260 | 88,874 | 36.81 | *aadE,, tet(M)* | *rplL* | TTATTTAAGAGTAAC | KX077888 |
| JH1308 | ST1 | 2 | Jiangsu, China | ICE*Ssu*JH1308-1 | 79,260 | 37.17 | *aph(3’)-III*, *sat4*, *aadE, erm(B), tet*(S) | In *SSU0468* | TTAATGACAG | KX077886 |
|  |  |  |  | ICE*Ssu*JH1308-2 | 75,677 | 37.45 | *aadE, tet(M)* | *rplL* | TTATTTAAGAGTAAC | KX077884 |
| JH1301 | ST383 | - | Jiangsu, China | ICE*Ssu*JH1301 | 81,649 | 37.75 | *aph(3')-III, erm(B), tet(O)* | *rplL* | TTATTTAAGAGTAAC | KX077887 |
|  |  |  |  | ΦJH1301-1 | 42,471 | 41.65 | - | *cysM* | AAAAAAACTATCTCGCCG | KX077891 |
|  |  |  |  | ΦJH1301-2 | 63,919 | 38.47 | *sat4*, *aadE, cat, mef(A)* | *rum* | CACATCGAGTGTGT | KX077896 |
|  |  |  |  | ΦJH1301-3 | 12,418 | 41.70 | - | *SSU1958* | ATACCATTTTGTGATA | KX077892 |
| LP081102 | ST1 | 1/2 | Zhejiang, China | ICE*Ssu*LP081102 | 64,515 | 38.25 | *erm(B), tet(O)* | *rplL* | TTATTTAAGAGTAAC | KX077885 |
|  |  |  |  | ΦLP081102 | 36,580 | 41.41 | - | tRNA-Arg | TCTCCCCTGCAGGAAT | KX077890 |
| ZJ20091101 | ST28 | 2 | Zhejiang, China | ICE*Ssu*ZJ20091101-1 | 69,397 | 38.98 | *erm(B), tet(O)* | In *SSU1262* | ATGACACTAT/ATGACACAAT | KX077882 |
|  |  |  |  | ICE*Ssu*ZJ20091101-2 | 112,763 | 38.38 | *tet(O)* | *rplL* | TTATTTAAGAGTAAC | KX077883 |
|  |  |  |  | ΦZJ20091101-1 | 32,297 | 40.82 | - | *cysM* | TTATATAGTAGA | KX077889 |
|  |  |  |  | ΦZJ20091101-2 | 9,473 | 36.84 | - | *SSU1320* | ACAACTTGAAAAAATAA | KX077893 |
|  |  |  |  | ΦZJ20091101-3 | 20,580 | 43.24 | - | *SSU0680* | - | KX077894 |
|  |  |  |  | ΦZJ20091101-4 | 39,766 | 41.59 | - | tRNA-Arg | TCTCCCCTGCAGGAAT | KX077895 |
| SC070731 | ST7 | 2 | Sichuan, China | CMGE_SC070731_ | 124,235 | 39.11 | *aph(3’)-III*, *sat4*, *aadE, erm(B)*, *mef(A)*, *msr(D)*, *tet(O)*, *tet(40)* | *rum* | CACGTG**GA**GTGTGT/CACATA**GA**AGTTGT | CP003922 |
| JS14 | ST7 | 14 | Jiangsu, China | CMGE_JS14_ | 124,457 | 39.13 | *aph(3’)-III*, *sat4*, *aadE, erm(B)*, *mef(A)*, *msr(D)*, *tet(O)*, *tet(40)* | *rum* | CACGTG**GA**GTGTGT/CACATA**GA**AGTTGT | CP002465 |
| D12 | ST619 | 9 | China | ICE*Ssu*D12 | 75,852 | 37.63 | *aph(3’)-III*, *sat4*, *aadE*, *erm(B)*, *tet(O)*, *tet(L)* | *rum* | CACGTG**GA**GTGTGT/CACATA**GA**AGTTGT | CP002644 |
| SsuD | ST1 | 2 | Italy | ΦSsuD.1 | 60,731 | 39.71 | *aph(3’)-III*, *sat4*, *aadE, erm(B)*, *tet(W)* | *rum* | CACGTG**GA**GTGTGT/CACATA**GA**AGTTGT | FN997652 |

^a^ Whole genome sequences of MGEs were closed by PCR

^b^ Insertion site genes were checked referring to P1/7.

^c^ Putative *att* sites were manually checked by compare of the junction sequences.
