# Supplementary figures and images for: Evolution and Diversity of the Antimicrobial Resistance Associated Mobilome in Streptococcus suis: A Probable Mobile Genetic Elements Reservoir for Other Streptococci
Source: Front Cell Infect Microbiol. 2016 Oct 7;6:118. doi: 10.3389/fcimb.2016.00118 (PMC5053989; doi:10.3389/fcimb.2016.00118)

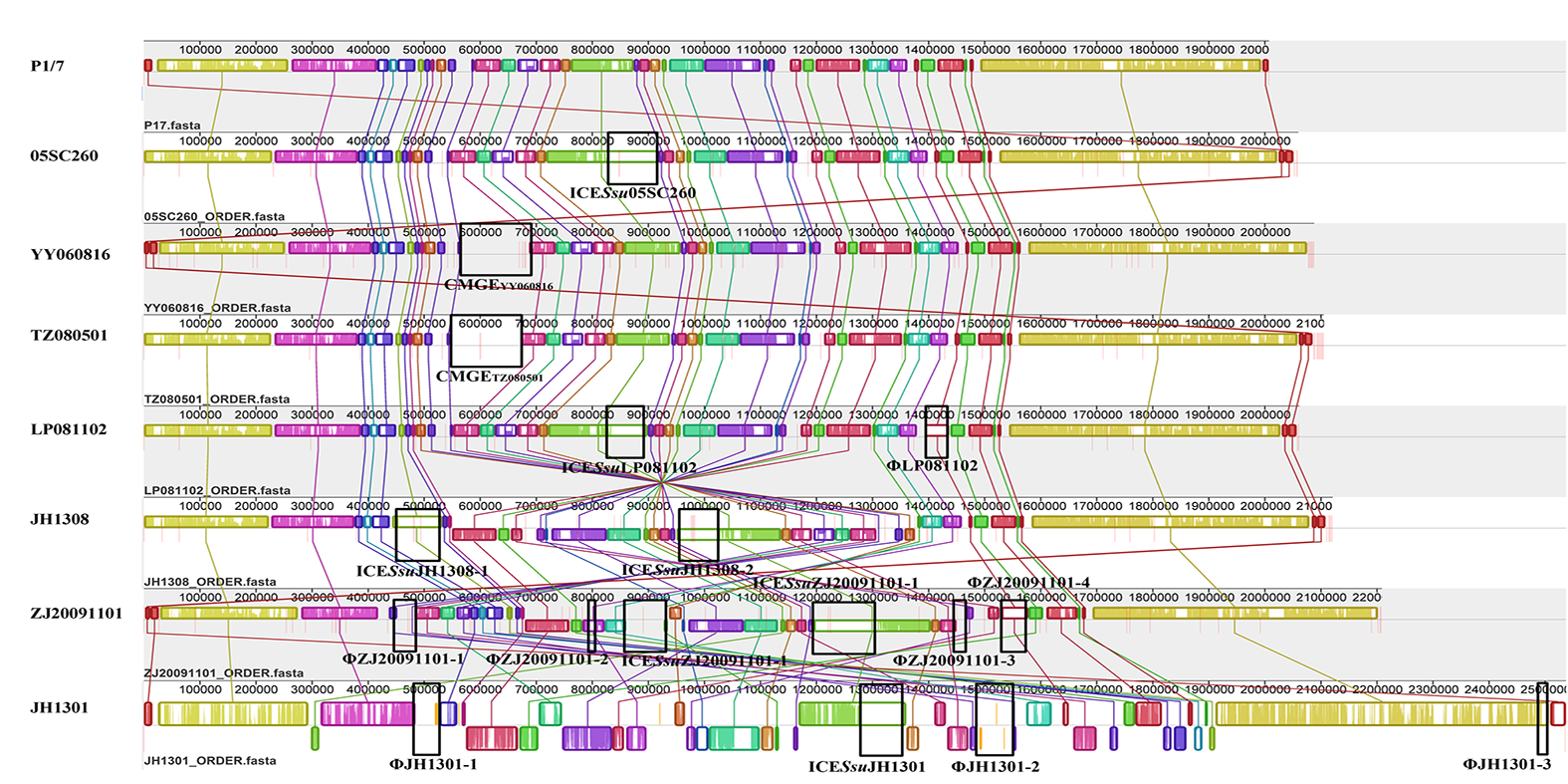

Supplement: Figure S1 — Comparative genomics of S.suis isolates and localization of MGEs in genomes. The scaffolds of each strains was ordered by MAUVE 2.4 with the whole genome sequenced reference strain including P1/7, 05ZYH33, BM407. The ordered genome sequences were further compared with P1/7. Black Box showed the MGEs after manually confirmed. [file Image1.TIF]

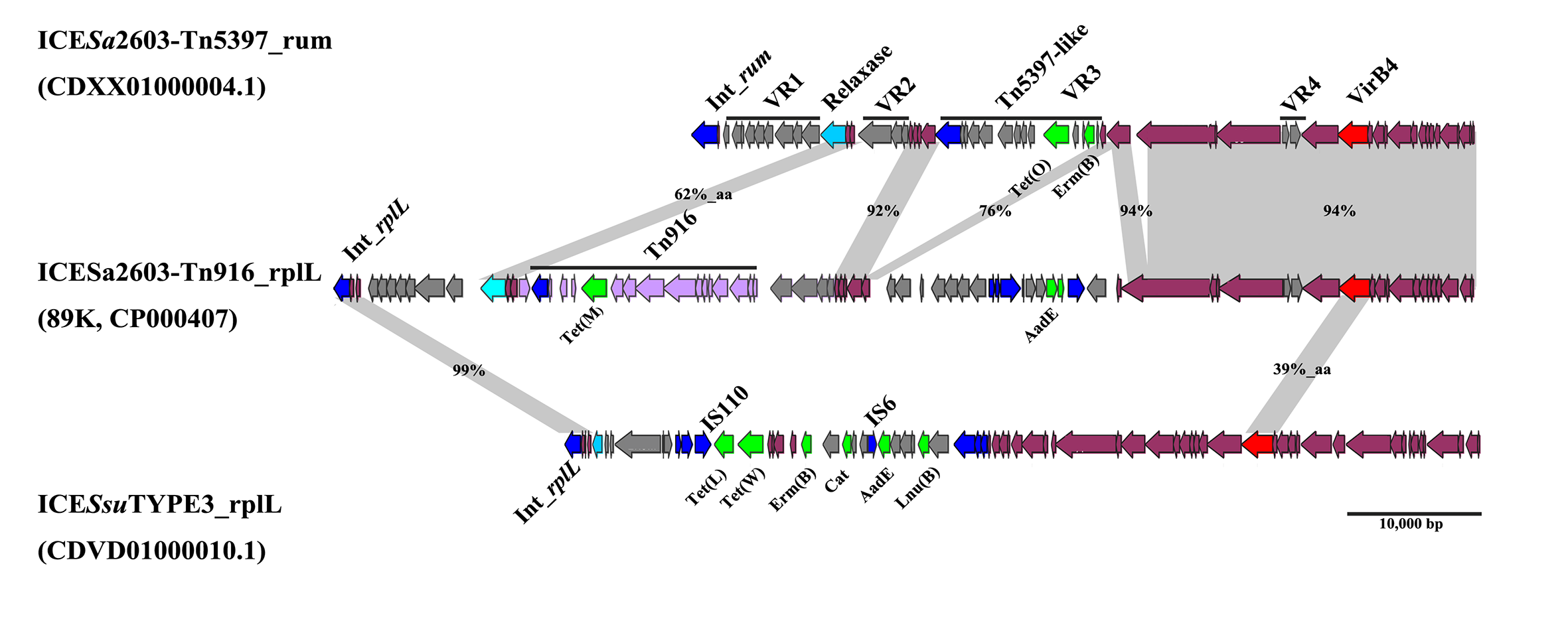

Supplement: Figure S2 — Schematic diagram of ICESa2603-Tn5397_rum, ICESa2603-Tn916_rplL, and ICESsuTYPE3_rplL. Int, relaxase, VirB4, and AMR determinants were indicated in blue, light blue, red, and green, respectively. ORFs identity was shown in light gray shadow and identity of amino acids (aa) was indicated. ICESa2603-Tn5397_rum and ICESa2603-Tn916_rplL showed encoding identical conjugation module but different integration module. ICESa2603-Tn916_rplL and ICESsuTYPE3_rplL had identical integration module but distinct conjugation module. [file Image2.TIF]

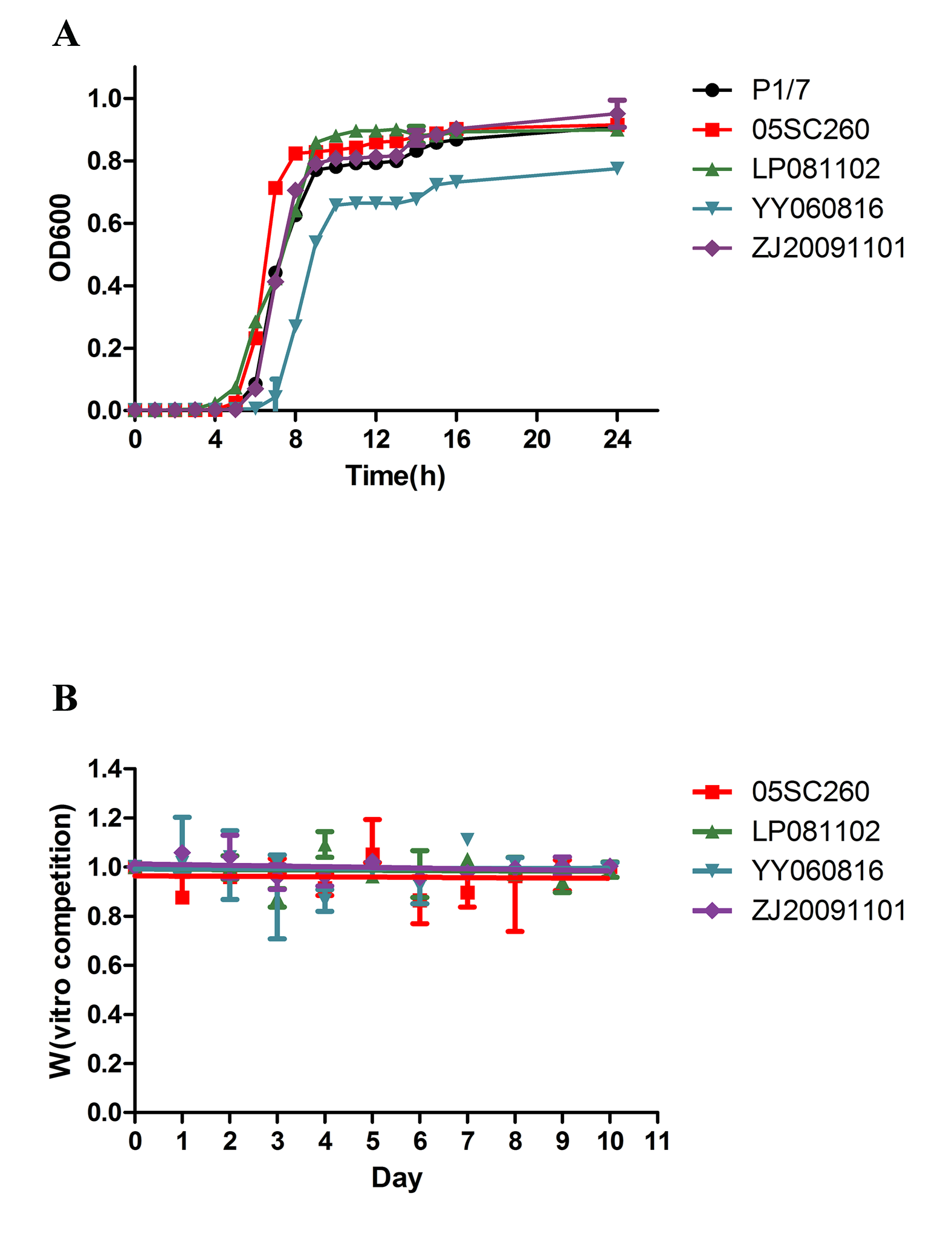

Supplement: Figure S3 — In vitro growth (A) and competition (B) of strain 05SC260, LP081102, YY060816, and ZJ20091101. Strain 05SC260 carry an ICESsu05SC260 which is nearly identical to 89K; LP081102 carry an ICE, ICESsuLP081102, and a prohage, ΦLP081102; YY060816 carry an ICE-phage tandem MGE, CMGEYY060816; and ZJ20091101 carry an ICE-ICE tandem MGE, ICESsuZJ20091101-2 and 5 other MGEs (See Table S1). [file Image3.TIF]
